# Insanity and Its Treatment: Lectures on the Treatment, Medical and Legal, of Insane Patients

**Published:** 1871-06

**Authors:** 


*Insanity and its Treatment: Lectures on the Treatment, Medical and Legal, of Insane Patients.* By G. FIELDING BLANDFORD, M.D., Fellow of the Royal College of Physicians, in London, etc., etc. *With a Summary of the Laws in force in the United States, on the Confinement of the Insane.* By ISAAC RAY, M.D.

---

The above work, of 465 pages, is published by Henry C. Lea, of Philadelphia, and issued from the press the present year.

It comprises twenty Lectures, and an appendix containing the laws of force in the several States, respecting the confinement of insane persons.

These Lectures are intended to instruct in the symptoms, pathology and treatment of insanity, without special reference to its connection with forensic medicine, they nevertheless, impress upon the reader those impoatant principles to be observed in affording medical testimony in courts of law.

A rational view is taken of the subject, and in the application of remedies for the treatment of the various forms of mental disease, the fact that the brain is the organ of the mind, and that the former is functionally or otherwise diseased when the latter is purturbated, is kept prominently in view.

Remedies, therefore, calculated to give increase of cerebral power, by direct stimulants, nourishment, etc., only hygenic and other indirect means, impressing the brain itself, are mentioned in connection with the treatment. We think the work a valuable addition to the library of a practicing physician.

